# BORN study: a multicenter randomized trial investigating cord blood red blood cell transfusions to reduce the severity of retinopathy of prematurity in extremely low gestational age neonates

**DOI:** 10.1186/s13063-022-06949-8

**Published:** 2022-12-13

**Authors:** Luciana Teofili, Patrizia Papacci, Nicoletta Orlando, Maria Bianchi, Tina Pasciuto, Iolanda Mozzetta, Fernando Palluzzi, Luciano Giacò, Carmen Giannantonio, Giulia Remaschi, Michela Santosuosso, Enrico Beccastrini, Marco Fabbri, Caterina Giovanna Valentini, Tiziana Bonfini, Eleonora Cloclite, Patrizia Accorsi, Antonella Dragonetti, Francesco Cresi, Giulia Ansaldi, Genny Raffaeli, Stefania Villa, Giulia Pucci, Isabella Mondello, Michele Santodirocco, Stefano Ghirardello, Giovanni Vento

**Affiliations:** 1 Fondazione Policlinico A. Gemelli IRCCS, Largo Gemelli 8, 00168 Rome, Italy; 2 Università Cattolica del Sacro Cuore, Rome, Italy; 3 Azienda Ospedaliero Universitaria Careggi, Florence, Italy; 4 Azienda Ospedaliero Universitaria Pisana, Pisa, Italy; 5 Azienda Sanitaria Locale-Presidio Ospedaliero di Pescara, Pescara, Italy; 6 Città della Salute e della Scienza, Turin, Italy; 7 Department of Public Health and Pediatrics, University of Turin, Turin, Italy; 8 Fondazione IRCCS Ca’ Granda Ospedale Maggiore Policlinico, Milan, Italy; 9 Department of Clinical Sciences and Community Health, University of Milan, Milan, Italy; 10 Azienda Ospedaliera Bianchi Melacrino Morelli, Reggio Calabria, Italy; 11 Ospedale Casa Sollievo della Sofferenza, San Giovanni Rotondo, Foggia, Italy; 12 Fondazione IRCCS Policlinico S. Matteo, Pavia, Italy

**Keywords:** Extremely low gestational age neonates, Retinopathy of prematurity, Transfusions, Fetal hemoglobin, Umbilical blood, Randomized controlled trial

## Abstract

**Background:**

Extremely low gestational age neonates (ELGANs, i.e., neonates born before 28 weeks of gestation) are at high risk of developing retinopathy of prematurity (ROP), with potential long-life visual impairment. Due to concomitant anemia, ELGANs need repeated red blood cell (RBC) transfusions. These produce a progressive replacement of fetal hemoglobin (HbF) by adult hemoglobin (HbA). Furthermore, a close association exists between low levels of HbF and severe ROP, suggesting that a perturbation of the HbF-mediated oxygen release may derange retinal angiogenesis and promote ROP.

**Methods/design:**

BORN (umBilical blOod to tRansfuse preterm Neonates) is a multicenter double-blinded randomized controlled trial in ELGANs, to assess the effect of allogeneic cord blood RBC transfusions (CB-RBCs) on severe ROP development. Recruitment, consent, and randomization take place at 10 neonatology intensive care units (NICUs) of 8 Italian tertiary hospitals. ELGANs with gestational age at birth comprised between 24+0 and 27+6 weeks are randomly allocated into two groups: (1) standard RBC transfusions (adult-RBCs) (control arm) and (2) CB-RBCs (intervention arm). In case of transfusion need, enrolled patients receive transfusions according to the allocation arm, unless an ABO/RhD CB-RBC is unavailable. Nine Italian public CB banks cooperate to make available a suitable amount of CB-RBC units for all participating NICUs. The primary outcome is the incidence of severe ROP (stage 3 or higher) at discharge or 40 weeks of postmenstrual age, which occurs first.

**Discussion:**

BORN is a groundbreaking trial, pioneering a new transfusion approach dedicated to ELGANs at high risk for severe ROP. In previous non-randomized trials, this transfusion approach was proven feasible and able to prevent the HbF decrease in patients requiring multiple transfusions. Should the BORN trial confirm the efficacy of CB-RBCs in reducing ROP severity, this transfusion strategy would become the preferential blood product to be used in severely preterm neonates.

**Trial registration:**

ClinicalTrials.gov NCT05100212. Registered on October 29, 2021

**Supplementary Information:**

The online version contains supplementary material available at 10.1186/s13063-022-06949-8.

## Introduction

Extremely preterm birth is associated with high mortality and adverse functional outcome [1]. Among diseases complicating the clinical course of premature birth, retinopathy of prematurity (ROP) oddly influences the neurodevelopmental outcome of affected patients [2]. ROP is one of the most important causes of childhood blindness [2, 3]. ROP develops in the immature retina as a consequence of an abnormal angiogenesis that follows the vasoconstriction caused by hyperoxia [4]. A body of evidence from retrospective and prospective studies suggest a connection between ROP, the number of red blood cell (RBC) units received, and the age at transfusion [5–11]. Usually, preterm infants are repeatedly transfused during hospitalization, with an average number ranging from 3 to 8 units in those born at age (GA) < 28 weeks [12, 13]. Conversely to non-transfused neonates, in whom fetal hemoglobin (HbF) persistently predominates over adult hemoglobin (HbA), in those receiving repeated transfusions, HbF progressively declines [14]. Despite HbF and HbA share similar three-dimensional structure, they deeply differ in chemical and physical properties. In particular, HbF exhibits higher oxygen affinity than HbA, more steadily maintains tetrameric integrity, and exerts a critical pseudo-peroxidase activity with a redox effect which neutralizes peroxides and removes radicals [15-17]. For premature neonates, with an immature enzymatic antioxidant system, HbF plays an essential role in the progressive adaptation to the postnatal oxygen-rich environment [18]. In addition, HbF generates unbound nitric oxide much faster than HbA, which, coupled with the high deformability of fetal RBC, assures the optimal perfusion in developing fetal tissues [19, 20].

### Rationale for transfusing cord blood

In neonates receiving RBC transfusions, the proportion of HbF over total Hb decreases proportionally to the pre-transfusion value [21]. Recently, low HbF has been associated with ROP severity. Stutchfield et al. (prospective study including 42 infants born at GA ≤ 32 weeks) reported a higher risk for severe ROP in neonates experiencing an average HbF level as low as 61.7%, until postmenstrual age (PMA) of 36 weeks [22]. Jiramongkolchai et al. (prospective study including 60 neonates born at GA ≤ 33 weeks) showed that neonates with HbF levels < 31.5% at PMA of 31 and 34 weeks had a risk of ROP higher 7.6 and 12.3 folds, respectively, than neonates who did not [23]. Hellström et al. (retrospective study including 385 infants born at GA ≤ 30 weeks) found that neonates developing any stage of ROP had equivalent HbF at birth but lower HbF levels in the first postnatal week [24]. The probability of ROP was about 60% in neonates with a mean HbF value of 40%, compared to only 10% probability in those with a mean HbF value of 90% [24]. RBC transfusions represent the mainstay for treating prematurity anemia and are unavoidable for most patients. Our group has been working for years on the hypothesis that transfusing RBCs collected from umbilical cord blood (CB-RBC) instead of blood from adult donors (A-RBC) might prevent the progressive loss of HbF and have a positive influence on the outcome of preterm neonates [25]. In 2014, we conducted a pilot non-randomized study assessing the feasibility of using RBC concentrates obtained from allogeneic umbilical blood to transfuse preterm neonates [26]. The units solidary donated at our cord blood bank that were unsuitable for transplant were fractionated and used for transfusing 20 neonates with GA at birth < 30 weeks. This study allowed us to conclude that transfusing CB-RBC concentrates is feasible and produces a similar hematocrit increment as standard transfusions [26]. We refined our fractionation methods [27], and in 2018, we performed a proof-of-concept study (CB-TrIP study, NCT 03764813) to demonstrate that CB-RBC transfusions effectively prevent HbF loss [28]. The study included 25 neonates born at GA ≤ 30 weeks: neonates were assigned to receive adult or CB-RBC products depending on the availability of an ABO-RhD-matched CB unit at first transfusion request. HbF was monitored three times a week and the primary outcome was the median value of HbF at PMA of 32 weeks. We demonstrated that transfusing CB-RBC maintains sustained HbF levels in patients receiving exclusively CB-RBCs and limits the HbF depletion in those receiving both CB-RBC and A-RBC units [28]. Moreover, median HbF values < 61.7% significantly predicted all-stage ROP [28].

### Hypothesis generation and study groundworks

Considering that ROP severity seems to be connected to low HbF levels and that CB transfusions prevent the HbF loss, it is plausible that transfusing RBC enriched with HbF instead of those containing HbA may impact positively over ROP development and progression. Umbilical blood is a source of hematopoietic stem cells and is collected by public CB banks (CBBs) from full-term neonates as a graft for transplanting hematological patients lacking a related donor. However, the content of hematopoietic stem cells is unpredictable, and most CB units are unsuitable for transplant and are recycled to research purposes or even disposed. Our previous studies on CB transfusion highlighted that it is challenging for a single CBB to provide an inventory of CB units adequate for a randomized sizeable trial. In the first pilot study, matched CB units were available in about 85% of transfusion events of RhD-positive neonates but in none of RhD-negative patients [26]. Similarly, in the CB-Trip study, most patients received both adult and CB units because CB units were not always available at the time of the blood product request [28]. Therefore, in a trial randomizing patients to adult donor or CB transfusions, the cooperation among public cord blood banks (CBBs) is pivotal to fulfil the transfusion need of patients assigned to the experimental arm.

## Materials and methods

### Study design

**Fig. 1:**
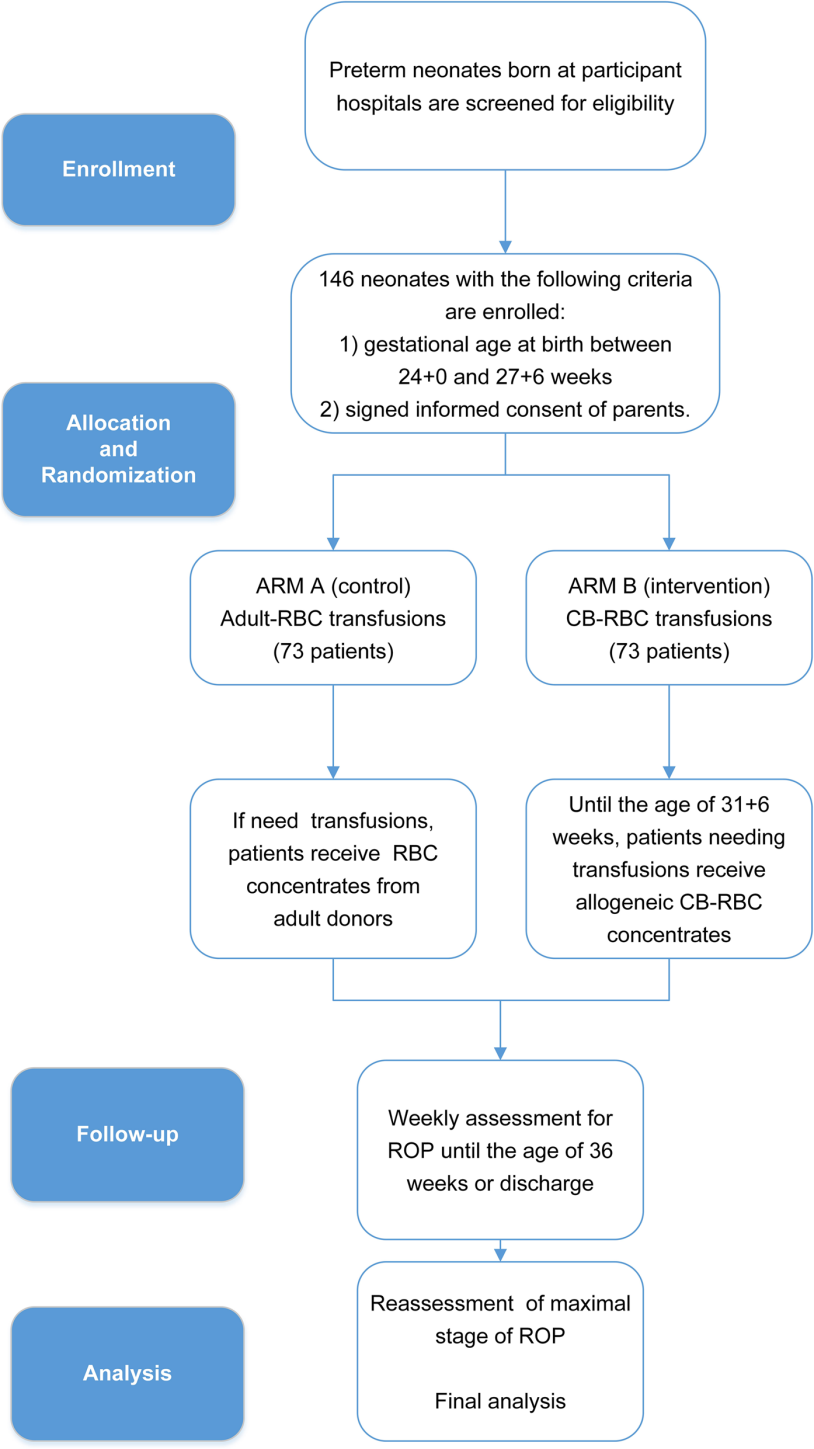
SPIRIT flow diagram of the study

BORN is a double-blind, multicenter, randomized (1:1) controlled trial that will assess whether transfusing CB-RBCs instead of adult-donor RBCs reduces the incidence of severe ROP (i.e., ROP stage 3 or higher). The study is no-profit, promoted by Fondazione Policlinico A. Gemelli and supported by Fresenius HemoCare (Italia). The protocol study received the approval of the Ethics Committee of Fondazione Policlinico A. Gemelli IRCCS in July 2021 (Study ID4364) and was registered at https://clinicaltrial.gov on October 29, 2021, with the identifier number NCT05100212 (URL https://clinicaltrials.gov/ct2/show/NCT05100212). The protocol was conceived following the Standard Protocol Items: Recommendations for Interventional Trials (SPIRIT) guidelines (Online supplement 1). Figure 1 depicts the SPIRIT flow diagram of the study. The main study characteristics have been illustrated in a section of the Fondazione Policlinico Gemelli IRCCS website, providing distinct information to health workers and relatives of preterm neonates (https://gemelligenerator.it/projects/).

### Sample size

The sample size has been calculated based on the primary outcome. Our recent observations suggest that high HbF values more than transfusion numbers are critical for developing severe ROP [28, 29]. For the sample size calculation, we considered data on severe ROP incidence recorded in the Vermont Oxford Network dataset, at our neonatal intensive care unit (NICU), and in the recent literature [30]. In the BORN study, we considered a significant reduction of severe ROP incidence after the treatment (primary endpoint), and therefore, the alternative hypothesis is as follows: incidence (severe ROP | A-RBC) > incidence (severe ROP | CB-RBC). In the study design, we accounted for three incidence values, according to different gestational age’s strata 38% (24 weeks), 20% (24–25 weeks), and 5% (26–27 weeks). In the treated branch, we expect a reduction of the proportion of cases to 10%, 5%, and 1% for the 24, 24–25, and 26–27 weeks stratum, respectively. Considering these three strata and the expected reduction in the treated sample per stratum, we estimated an effect size for the proportion difference of $h=2\arcsin \left(\sqrt{p_0}\right)-2\arcsin \left(\sqrt{p_1}\right)$ = 0.5, corresponding to a moderate effect size according to Cohen [31], where *p*_0 and *p*_1 are the marginal proportions of severe ROP cases in the untreated and treated subjects, respectively. Without considering multi-centricity, with a significance level of 0.05, and a power of 0.8, a sample size of *n* = 2 × 31.3 ≈ 63 subjects would be sufficient to detect a moderate effect (*h* = 0.5). Considering a mortality rate of 15% during the study period, *n* is increased to 2 (31.3 + 0.15 × 31.3) ≈ 72 subjects. Including the random effect due to the centers involved in this study, using a Cochran-Mantel-Haenszel test [32], with the same proportions, significance level, and power as the previous test, we calculated a total sample size of 146 subjects (73 per arm).

### Study population

A total of 146 extremely low gestational age neonates (i.e., neonates born < 28 gestation weeks, ELGAN) will be consecutively enrolled. Patient screening will occur soon after delivery or at NICU admission and will be carried out by neonatologists. Inclusion criteria are gestational age (GA) at birth between 24+0 and 27+6 weeks and signed informed consent of parents. Exclusion criteria are one or more of the following: maternal-fetal immunization, hydrops fetalis, major congenital malformations associated or not with genetic syndromes, previous transfusions, hemorrhage at birth, congenital infections, out-born infants, and health care team deeming it inappropriate to approach the infant’s family for informed consent. Umbilical cord clamping and cardiopulmonary resuscitation are managed according to the American Heart Association guidelines [33], starting oxygen delivery at a lower concentration (21–30%), and using pulse oximetry for subsequent titration [34]. Patients enrolled in this study receive equivalent standard therapies (i.e., treatments recommended in protocols in use at each center) as other patients. These may include erythropoietin administration, transfusion thresholds, and O_2_ therapy management. The O_2_ saturation target in enrolled patients is evaluated according to the PMA and is not superior to 95%. The enrollment of participants in other interventional trials is not allowed.

### Participating sites and the role of the Italian cord blood Bank network

**Fig. 2:**
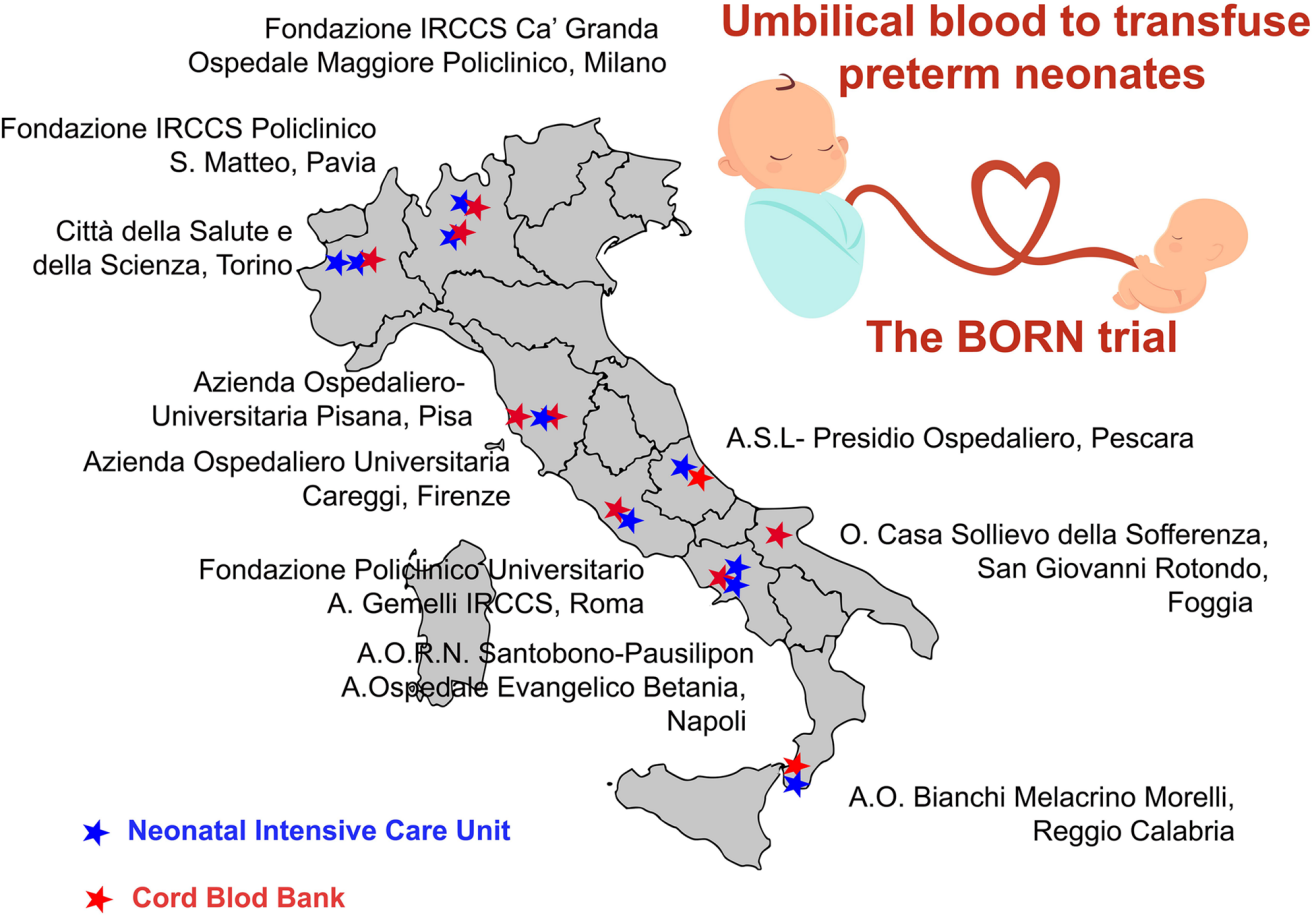
Map of cord blood banks and neonatal intensive care units participating to the BORN study

Patients are recruited at 10 different neonatology intensive care units (NICUs). In parallel, 10 CBBs provide NICUs with adequate CB units (Fig. 2). According to national regulation, Italian CBBs belong to a unique public network (Italian Cord Blood Bank Network), share homogeneous procedures on the quality and safety of blood products, and are functional units of public blood banks. This allows that participating CBBs can produce and distribute CB-RBC concentrates over the entire national area.

### Randomization and concealment

Patients are randomized 1:1 to receive standard A-RBC transfusions (arm A, comparator) or CB-RBC transfusions (arm B, intervention). After completing 31 weeks of PMA (31+6), all patients receive A-RBCs. All assigned RBC units are ABO/RhD matched; if an ABO/RhD-matched CB-RBC unit is unavailable, patients in arm B receive A-RBCs. Randomization sequences are generated by T.P. according to a restricted randomization procedure [35]. The allocation sequence is generated using both stratification and permuted blocks with random block sizes and block order using the NCSS 2020 Statistical Software (2020), NCSS, LLC. Kaysville, UT, USA, ncss.com/software/ncss. Stratification factors are center and gestational age (< or ≥ 26 weeks, given the higher incidence of ROP in neonates born before 26 weeks). Twins are assigned to the same arm. The allocation table is not disclosed to ensure concealment, and the randomization is provided through Research Electronic Data Capture (RedCap) web application hosted at Fondazione Policlinico Gemelli IRCCS [36]. Randomization is performed by the medical staff at the blood bank: the medical staff involved in the assessment of clinical outcomes remains unaware of which arm the neonates are assigned to, until research is completed. To conceal the type of blood product, A-RBCs and CB-RBCs are distributed to NICUs in the same type of bags (CompoFlex 4F RCC storage pediatric bags).

### Intervention

CB-RBCs (intervention) consist of filtered leukodepleted, irradiated RBC concentrates obtained from umbilical CB. CB units are first leukodepleted through BioR Flex transfusion filters (Fresenius HemoCare) and then fractionated using cell separator CompomatG5 (Fresenius Kabi). RBCs are recovered, suspended in saline adenine glucose-mannitol (SAG-M) additive solution, and stored in CompoFlex 4F RCC storage pediatric bags not made with di (2-ethylhexyl) phthalate (DEHP). Fresenius HemoCare kindly provides cell separator, filters, bags, and SAG-M. CB transfusions are subjected to the same screening for transfusion-transmitted infectious disease as standard A-RBC transfusions (serology and nucleic acid tests for HIV, HBV and HCV, syphilis test, and other seasonal infectious markers in accordance with the Italian law). In addition, CB units are tested for fungal and bacterial cultures. As A-RBC units, all CB-RBCs are γ-irradiated at distribution and transfused within 24 h from irradiation. Transfusion therapy is managed according to center procedures, including transfusion triggers and infusion time.

### Study outcomes

The primary outcome is the incidence of severe ROP (stages 3 or higher) in CB-RBC and A-RBC arms at discharge or 40 weeks of PMA, which occurs first. Diagnosis and staging of ROP are carried out according to the International Classification, based on an ordinal scale with higher numbers indicating a more severe outcome: 0.1.2.3.3+, 4, and 5.1 [37]. ROP diagnosis will be made by experienced ophthalmologists, through indirect ophthalmoscopy and serial RetCam imaging. At the same endpoint of ROP, the following secondary outcomes are evaluated: incidence of ROP requiring treatment (laser therapy or anti-VEGF administration), incidence of bronchopulmonary dysplasia (BPD) [1, 38], and incidence of a composite outcome including any of the following: death, severe ROP, BPD, and necrotizing enterocolitis (NEC) [39, 40]. Additional laboratory outcomes are the median HbF level predicting severe ROP and BPD, intervals between consecutive transfusions, and hematocrit increase after transfusion.

### Data and safety monitoring board

The data and safety monitoring board (DSMB) is represented by the local COBUS (committee for the proper use of blood). According to the Italian regulation, COBUS must operate in each hospital to plan and monitor medicine transfusion activities. COBUS of Fondazione Policlinico A. Gemelli IRCCS will assess clinical study progress and safety data. Moreover, it will provide the sponsor with recommendations regarding study modification, continuation, or termination.

### Adverse events

Due to the nature of the investigated product (cord blood), adverse events related to transfusions are managed according to the Italian regulation on transfusion surveillance. Data are recorded in the dedicated section of “Sistema Informativo dei Servizi Trasfusionali – SISTRA,” instituted on December 21, 2007, by the Italian Ministry of Health. The study start has been notified to the national transfusion surveillance committee, who has the authority to stop the trial early if patient safety is compromised.

### Interim analysis

An interim analysis has been planned with a twofold objective to confirm the safety of CB-RBC transfusions and evaluate the planned sample size. For these purposes, we scheduled a preliminary analysis when the first 58 enrolled patients are evaluable for the primary outcome. This estimation was performed considering the above reported reduction of the ROP incidence in different patient strata and applying a proportion test with fixed *h* = 0.5, significance level of 0.1, and power of 0.8 [41]. This resulted in a sample size of *n* = 2 × 25 = 50 neonates (25 per arm) which was further increased to 58 total patients (29 per arm) considering a mortality rate of 0.15. When 58 patients have been discharged or have reached the 40° week of PMA (which occurs first), data will be analyzed by an independent third party (a scientist from the Bioinformatics team of Fondazione Policlinico A. Gemelli IRCCS who are not directly involved in the study). The analysis report will be examined by the DSMB (i.e., COBUS of Fondazione Policlinico A. Gemelli IRCCS). If no severe adverse events related to the treatment occurred and data suggested a clinical response that warrants further investigation, the study will proceed. Moreover, should data raise doubt on the dependability of the planned sample size, this will be adjusted to ensure the desired study power [42]. Finally, the study duration will be revaluated if it is deemed necessary on the basis of the results of the interim analysis.

### Data management

Two different types of electronic case report form (eCRF) have been designed, using REDCap (RRID:SCR_003445), an electronic data capture hosted at Fondazione Policlinico Universitario A. Gemelli, IRCCS (https://redcap-irccs.policlinicogemelli.it/). This is a secure, web-based application designed to support data capture for research studies, providing (1) an intuitive interface for validated data entry, (2) audit trails for tracking data manipulation and export procedures, (3) automated export procedures for seamless data downloads to common statistical packages, and (4) procedures for importing data from external sources [36, 43]. Access to the data will be allowed through authentication and authorization systems exclusively to study investigators. Furthermore, to protect the identity of participants, pseudonymization techniques will be adopted to make the data directly attributable to patients. Finally, data will be processed exclusively by personnel authorized for this purpose and subject to professional secrecy and the legal obligation of confidentiality, in compliance with the protection of the rights and dignity of the patient. The eCRFs for epidemiological, clinical, and laboratory data relative to enrolled patients are accessible only to NICU investigators, except for the randomization section that can be accessed only by blood bank investigators.

Table 1 shows data recorded in neonates enrolled in the trial. In addition, CBB investigators report another type of eCRFs for the data relative to the processing of CB units, including complete cell blood count (CBC) before and after fractionation, residual leukocytes after filtration, hematocrit at distribution, and free hemoglobin at the end of the storage.

**Table 1:** Overview of the schedule of assessments planned in the BORN study

Postmenstrual age (week)		First week							Second week							PMA weeks 3 to 35	PMA week 36	PMA weeks 37 to 40
Postnatal day	Baseline	1	2	3	4	5	6	7	8	9	10	11	12	13	14			
Informed consent^a^	●																	
Confirmation of study eligibility	●																	
Demography	●																	
Pregnancy history^b^	●																	
Physical examination^c^	●																	
Birth weight	●																	
Body weight	●	●	●	●	●	●	●	●	●	●	●	●	●	●	●	●	●	●
Length	●																	
Head circumference	●																	
Blood group	●																	
Iatrogenic blood loss	●	●	●	●	●	●	●	●	●	●	●	●	●	●	●	●	●	●
Respiratory support	●	●	●	●	●	●	●	●	●	●	●	●	●	●	●	●	●	●
Nutritional support		●	●	●	●	●	●	●	●	●	●	●	●	●	●	●	●	●
Brain ultrasound for IVH		●		●			●											
Echocardiography for PDA			●					●										
Hematological assessment^d^	●	● ●							●●							●● W	●●	●● W
IV access status	●	●							●							● W	●	● W
Concomitant therapies		●							●							● W	●	● W
RBC transfusion^e^		● (if any)^e^							● (if any)^e^							● (if any)^e^	● (if any)^e^	● (if any)^e^
Adverse event^f^		● (if any)^f^							● (if any)^f^							● (if any)^f^	● (if any)^f^	● (if any)^f^
ROP^g^																● W (from w 29)^g^	●	● W
BPD																	●	● W

*RBC* red blood cell, *ROP* retinopathy of prematurity, *BPD* bronchopulmonary dysplasia, *IVH* intraventricular hemorrhage, *CRF* case report form, *W* a week

^a^Informed consent must be signed and dated both by subject’s parent(s)/legally authorized representative(s) or the birth mother prior to any study-related procedures

^b^Pregnancy history includes information regarding placental abruption, placenta previa, chorioamnionitis, eclampsia, preeclampsia, diabetes, fetal Doppler velocimetry, fetal growth restriction, premature rupture of membranes, and pre-birth treatment (e.g., magnesium sulfate therapy, steroid treatment, or antibiotic treatment)

^c^Baseline physical examination and risk assessment include APGAR scores at 1 and 5 min and clinical risk index for babies II score

^d^Hematological assessment includes total Hb (g/dl), fetal Hb (%), and hematocrit (%)

^e^RBC transfusion must be recorded in CRF each time they are administered; identification code number, volume, and hematocrit of RBC unit must be reported; post-transfusion data include newborn hematocrit, pH, lactate, and potassium

^f^Adverse events include information regarding infections and neurological, cardiovascular, pulmonary, gastrointestinal, renal, and surgical diseases. Adverse events related to transfusions are notified to the Italian hemovigilance committee

^g^The first ROP exam will be performed at 29–30 weeks postmenstrual age. Subsequent ROP examination should be conducted weekly, or more frequently, based on clinical ophthalmological evaluation

### Protocol deviations

Non-compliant patients. Non-compliance to the assigned intervention (adult blood or cord blood RBC units) might occur due to the unavailability of CB ABO/RhD-matched units in single centers. According to our previous experiences, this event might occur particularly for Rh-negative patients [26, 28]. To limit the non-compliance rate, blood bank departments can access the entire inventory of CB units, which can be relocated according to the center needs.

### Statistical analysis

Based on the design mentioned above, the comparison of the proportion of patients free from severe ROP and other comorbidities is made between A versus B through the Cochran-Mantel-Haenszel test. The best cut point value of HbF (expressed as the median value of repeated measures collected from birth to PMA of 32 or 36 weeks) predicting ROP or other outcomes will be identified by the AUROC (area under the receiver operating characteristics) analysis. The association between HbF level and risk for prematurity-associated diseases (ROP, BPD, NEC, IVH) will be investigated by logistic regression analysis and expressed as an odds ratio with a relative 95% confidence interval (95% CI). Co-interventions like erythropoietin administration, transfusion thresholds, and O_2_ therapy will be included among covariates. The interval between two consecutive transfusions and hematocrit increase after CB-RBC or A-RBC transfusions in the two arms are compared by the Mann-Whitney *U* test. The following sets of analysis are planned: (1) all enrolled patients, independently if they were or not transfused; (2) all enrolled patients receiving transfusions, including major protocol deviations (i.e., patients in the CB-RBC arm receiving one or more A-RBC transfusions due to CB-RBC transfusion unavailability); and (3) all enrolled patients receiving transfusions, excluding major protocol deviations (i.e., patients in the CB-RBC arm receiving one or more A-RBC transfusions due to CB-RBC transfusion unavailability).

## Discussion

The effect of preterm birth among survivors may continue throughout life, impairing neurodevelopmental functioning, causing learning impairment, and affecting long-term physical health with a higher risk of non-communicable disease [44]. ROP, for example, determines lifelong visual disability in 3% of born before 32 weeks of gestation [3]. Long-term disabilities due to preterm birth represent an unmet need and convey a heavy burden to families, society, and the health system. Although the connection between preterm comorbidities and transfusions seems undisputable, two recent sizeable trials comparing the impact of large or restrictive transfusion thresholds on preterm neonate outcomes failed to evidence significant differences in major comorbidities [45, 46]. More severely ill neonates are likely to receive more transfusions, and it is extremely arduous to decipher the detrimental effects of blood products. The BORN trial specifically investigates the harmful effect due to the loss of physiological levels of fetal hemoglobin. Our approach is highly attractive, allowing to expand present knowledge on the detrimental effects of transfusions. Suppose the BORN trial demonstrates the efficacy of CB-RBC in limiting ROP severity, this groundbreaking and sustainable therapeutic approach might be rapidly implemented in every blood bank, including low-income countries. From the side of CBBs, not only is this strategy extremely low-expensive, but recycling the units that are not suitable for transplantation may in part balance the costs currently faced for CB unit collection.

## Trial status

The BORN trial (protocol version 5, August 5, 2021) is presently ongoing. Recruitment started in December 2021 and will be approximately completed in December 2023.

## Supplementary Information

**Additional file 1:.** SPIRIT checklist.

## Abbreviations

A-RBC: Adult-RBC
BPD: Bronchopulmonary dysplasia
CB-RBC: Cord blood-RBC
CBB: Cord blood bank
DEHP: (2-Ethylhexyl) phthalate
ELGAN: Extremely low gestational age neonate
GA: Gestational age
HbA: Adult hemoglobin
HbF: Fetal hemoglobin
HBV: Hepatitis B virus
HCV: Hepatitis C virus
HIV: Human immunodeficiency virus
IVH: Intraventricular hemorrhage
NEC: Necrotizing enterocolitis
NICU: Neonatal intensive care unit
PMA: Postmenstrual age
RBC: Red blood cell
ROP: Retinopathy of prematurity
SAG-M: Saline adenine glucose-mannitol
